# Membrane Permeabilization and Antimicrobial Activity of Recombinant Defensin-d2 and Actifensin against Multidrug-Resistant *Pseudomonas aeruginosa* and *Candida albicans*

**DOI:** 10.3390/molecules27144325

**Published:** 2022-07-06

**Authors:** Ifeoluwa D. Gbala, Rosaline W. Macharia, Joel L. Bargul, Gabriel Magoma

**Affiliations:** 1 Molecular Biology and Biotechnology, Institute for Basic Sciences, Technology and Innovation, Pan African University, Nairobi P.O. Box 62000-00200, Kenya; gmagoma@jkuat.ac.ke; 2 Centre for Biotechnology and Bioinformatics, University of Nairobi, Nairobi P.O. Box 30197-00100, Kenya; rosaline@uonbi.ac.ke; 3 Department of Biochemistry, Jomo Kenyatta University of Agriculture and Technology, Nairobi P.O. Box 62000-00200, Kenya; jbargul@icipe.org; 4 International Centre of Insect Physiology and Ecology, Nairobi P.O. Box 30772-00100, Kenya

**Keywords:** recombinant, antimicrobial peptides, spinach defensin, actifensin, multidrug-resistant

## Abstract

Antimicrobial resistance requires urgent efforts towards the discovery of active antimicrobials, and the development of strategies to sustainably produce them. Defensin and defensin-like antimicrobial peptides (AMPs) are increasingly gaining pharmacological interest because of their potency against pathogens. In this study, we expressed two AMPs: defensin-d2 derived from spinach, and defensin-like actifensin from *Actinomyces ruminicola*. Recombinant pTXB1 plasmids carrying the target genes encoding defensin-d2 and actifensin were generated by the MEGAWHOP cloning strategy. Each AMP was first expressed as a fusion protein in *Escherichia coli*, purified by affinity chromatography, and was thereafter assayed for antimicrobial activity against multidrug-resistant (MDR) pathogens. Approximately 985 µg/mL and 2895 µg/mL of recombinant defensin-d2 and actifensin, respectively, were recovered with high purity. An analysis by MALDI-TOF MS showed distinct peaks corresponding to molecular weights of approximately 4.1 kDa for actifensin and 5.8 kDa for defensin-d2. An in vitro antimicrobial assay showed that MDR *Pseudomonas aeruginosa* and *Candida albicans* were inhibited at minimum concentrations of 7.5 µg/mL and 23 µg/mL for recombinant defensin-d2 and actifensin, respectively. The inhibitory kinetics of the peptides revealed cidal activity within 4 h of the contact time. Furthermore, both peptides exhibited an antagonistic interaction, which could be attributed to their affinities for similar ligands, as deduced by peptide–ligand profiling. Moreover, both peptides inhibited biofilm formation, and they exhibited no resistance potential and low hemolytic activity. The peptides also possess the ability to permeate and disrupt the cell membranes of MDR *P. aeruginosa* and *C. albicans*. Therefore, recombinant actifensin and defensin-d2 exhibit broad-spectrum antimicrobial activity and have the potential to be used as therapy against MDR pathogens.

## 1. Introduction

Antimicrobial resistance is a public health threat, with the potential to cause mortalities estimated at 1 million per annum by 2050 if new effective antimicrobials are not developed [1]. The mortality rates of infections associated with multidrug-resistant (MDR) microorganisms have consistently increased over the last two decades across different populations [2]. This creates an urgent situation for the development of effective alternatives, or the repurposing of existing ones. In line with this, antimicrobial peptides (AMPs) are currently being explored as pharmacologically important alternatives [3]. AMPs represent a part of the innate immune systems in almost all classes of life, including microorganisms, plants, and animals [4]. They exhibit a great number of fundamentally different functional activities, which implies that there is no single drug-target site or mechanism of action that is common to all AMPs [5]. The crude isolation of AMPs from their natural sources has been achieved by previous studies. However, this involves a laborious process that yields products of low purity and quantity [4]. The alternative approach of the chemical synthesis of peptides is limited by the high costs of production [6], which are unsustainable. Therefore, the recombinant production of AMPs offers an appealing strategy because of the ease of upscaling, or optimization, and the time and cost effectiveness.

Plant defensins are small (12–58 amino acids; 5–7 kDa) cationic highly basic peptides that contain 8–10 cysteine residues that form 3–4 disulfide bridges for molecular stability [7]. Defensin-d2 is a plant defensin that is isolated from the leaves of *Spinacia oleracea* (spinach) [8]. The bioactivity of defensin-d2 against a broad range of phytopathogens, including *Pseudomonas syringae*, *Ralstonia solanacearum* and *Fusarium culmorum* [8,9], has been established. Actifensin is a defensin-like bacteriocin that is produced by *Actinomyces ruminicola*, which is also cysteine-rich and contains disulfide bonds [10]. Actifensin showed remarkable antibacterial activity against Gram-positive bacteria, including methicillin-resistant *Staphylococcus aureus*, but its antifungal activity is yet to be established [10]. While plant AMPs often exert broad-spectrum inhibitory actions at high concentrations, the bacterial AMPs are reported to exhibit narrow-spectrum actions at lower concentrations [8,9,10,11,12,13]. Thus, the exploration of the individual and synergistic antimicrobial activities of AMPs is important for antimicrobial repurposing.

Sustainable strategies for the large-scale production of AMPs for pharmaceutical applications are currently of high priority [14]. A promising method that is scalable for the production of AMPs with high purities and yields is the recombinant expression of the AMPs in *Escherichia coli*, because of its unique advantages [14,15]. However, various challenges face the recombinant expression of AMPs in *E. coli*, which include codon bias, the toxicity of proteins to the expression host, and the inability to process posttranslational modification, which is crucial for the correct protein folding and efficient functionality of the recombinant protein. Fusion expression, however, has been suggested as an effective strategy to reduce the toxic effect of AMPs on the host cells, and it also shields the AMPs from proteolytic degradation [16]. Therefore, this study sought to express defensin-d2 and actifensin as recombinant fusion proteins in *E. coli*, and to investigate the antimicrobial actions of the purified recombinant peptides against selected multidrug-resistant pathogens. This study also evaluated the cell-permeability potential of the recombinant peptides, as a possible mechanism of action.

## 2. Results

### 2.1. Bioinformatics Analysis of Defensin-d2 and Actifensin

The amino acid sequences of defensin-d2 and actifensin, as well as their physicochemical properties, are presented in Table 1. On the one hand, defensin-d2 is composed of 52 amino acids, has a calculated molecular weight of 5803.73 Da and a net charge of +7.6 at a pH of 7.0. The peptide has an isoelectric point (pI) of 9.3, an average hydrophilicity of 0.5 (about a 44% ratio of hydrophilic residues to total number of residues) and a GRAVY score of −0.810. On the other hand, actifensin is composed of 37 amino acids, has a calculated molecular weight of 4097.70 Da and a net charge of +3.8 at a pH of 7.0. Actifensin has a pI of 8.89, an average hydrophilicity of −0.4 (about a 30% ratio of hydrophilic residues to total number of residues) and a GRAVY score of −0.243. The instability and aliphatic indices of the peptides indicated that actifensin is more stable (II = 8.22; AI = 47.30) compared with defensin-d2 (II = 55.68; AI = 24.42). The disulfide bonds in defensin-d2 were predicted to have cysteine connectivity patterns of Cys1–Cys8, Cys2–Cys5, Cys3–Cys6 and Cys4–Cys7, while, for actifensin, they were predicted as Cys1–Cys4, Cys2–Cys3 and Cys5–Cys6.

### 2.2. Generation of Genes and Recombinant Plasmids

**Figure 1:**
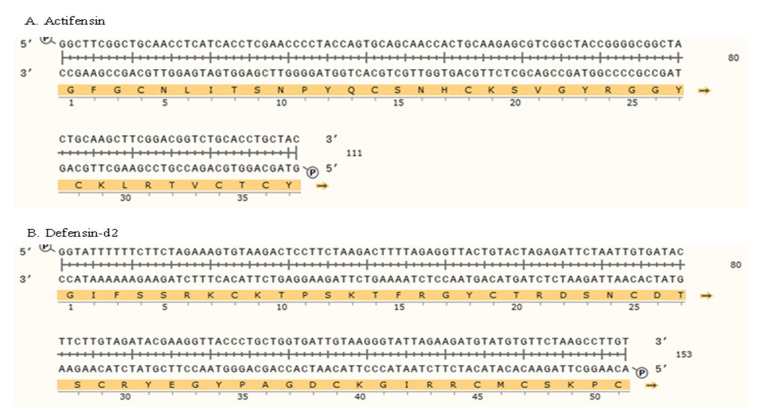
Schematic representation of gene constructs of ORFs encoding (**A**) actifensin and (**B**) defensin-d2, optimized to *E. coli* codon usage. Construct was generated using Snapgene v 1.1.3 (from Insightful Science; available at snapgene.com).

**Figure 2:**
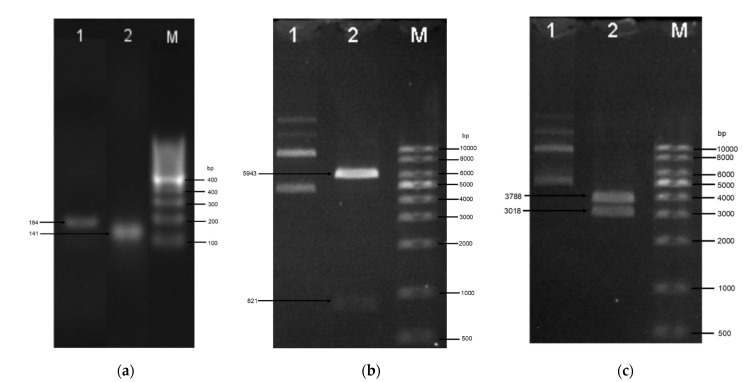
Agarose gel electrophoresis of (**a**) amplification of the target genes and (**b,c**) recombinant plasmids. (**a**) Target genes flanked by homologous sequences upstream and downstream of the MCS of pTXB1. L1—defensin-d2; L2—actifensin; M—100 bp ladder. (**b**) Recombinant actifensin-pTXB1 generated using MEGAWHOP cloning. L1—circularized recombinant actifensin-pTXB1; L2—recombinant plasmid digested with HindIII and BamHI; M—1 kb ladder. (**c**) Recombinant defensin-d2-pTXB1 generated using MEGAWHOP cloning. L1—circularized recombinant defensin-d2-pTXB1; L2—recombinant plasmid digested with EcoRV and BamHI; M—1 kb ladder.

The amino acid sequences of the peptides were reverse-translated to generate the open reading frames encoding the defensin-d2 and actifensin peptides. The ORFs generated were 111 bp and 153 bp fragments for actifensin and defensin-d2, respectively, and they were optimized according to the *E. coli* codon usage (Figure 1). The gene constructs were amplified as fragments of 141 bp and 183 bp for actifensin and defensin-d2, respectively, and they were flanked by 30 bp of sequences that were homologous upstream and downstream of the pTXB1 multiple cloning sites (MCSs) (Figure 2a). The recombinant plasmids, pTXB1-defensin-d2 and pTXB1-actifensin, generated through MEGAWHOP PCR, were resolved on 0.8% agarose gel as products of about 6806 bp and 6764 bp, respectively (Figure 2b,c). The conformation of the bands on the gel showed a successful circular amplification that was facilitated by the homologous recombination of the amplified target genes to the pTXB1 plasmid.

After the transformation, the presence of the target genes and plasmid were detected in positive clones as products of 102 bp, 103 bp or 730 bp for defensin-d2, actifensin and pTXB1, respectively (Figure S1). An analysis of the reads from the plasmid sequencing showed that both target genes were fused in-frame with the start codon and fusion partners, and in the correct orientation on the plasmid, without the incorporation of unwanted nucleotides (Figure S2).

### 2.3. Expression and Purification of Recombinant Defensin-d2 and Actifensin

**Figure 3:**
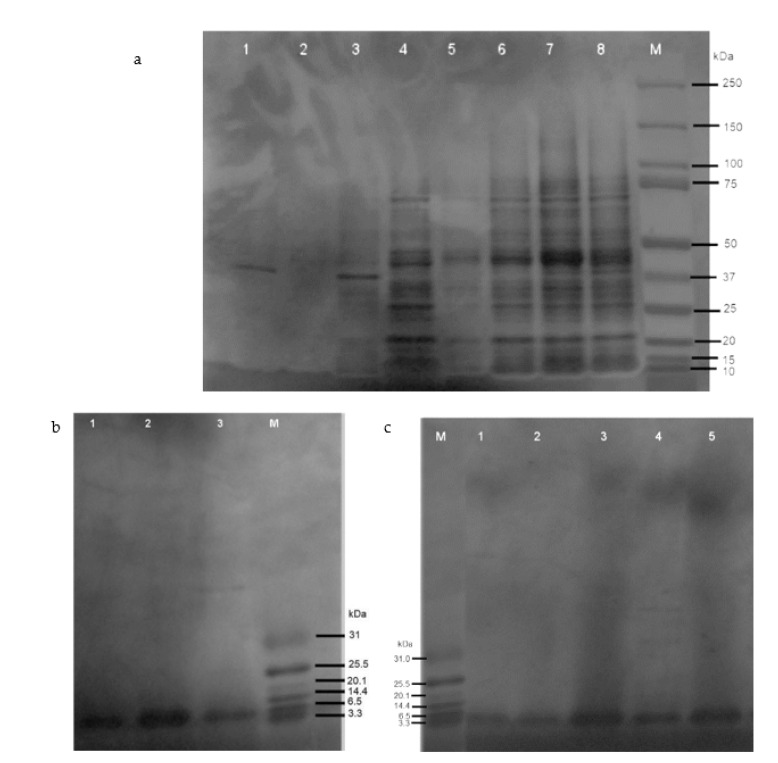
SDS-PAGE analysis of (**a**) recombinant fusion proteins and (**b,c**) purified recombinant peptides: * C1—15 °C for 18 h; C2—30 °C for 4 h; C3—37 °C for 2 h. (**a**) Expressions of recombinant fusion proteins at different induction conditions: L1—uninduced defensin-d2; L2—uninduced actifensin; L3—induced defensin-d2 at C1; L4—induced actifensin at C1; L5—induced defensin-d2 at C3; L6—induced actifensin at C3; L7—induced defensin-d2 at C2; L8—induced actifensin at C2; M—250 kDa protein marker. (**b**) Purified recombinant defensin-d2 after cleavage of fusion partner and affinity chromatography purification: L1—3; M—31 kDa ladder. (**c**) Purified recombinant actifensin after cleavage of fusion partner and affinity chromatography purification: L1—3; M—31 kDa ladder.

**Figure 4:**
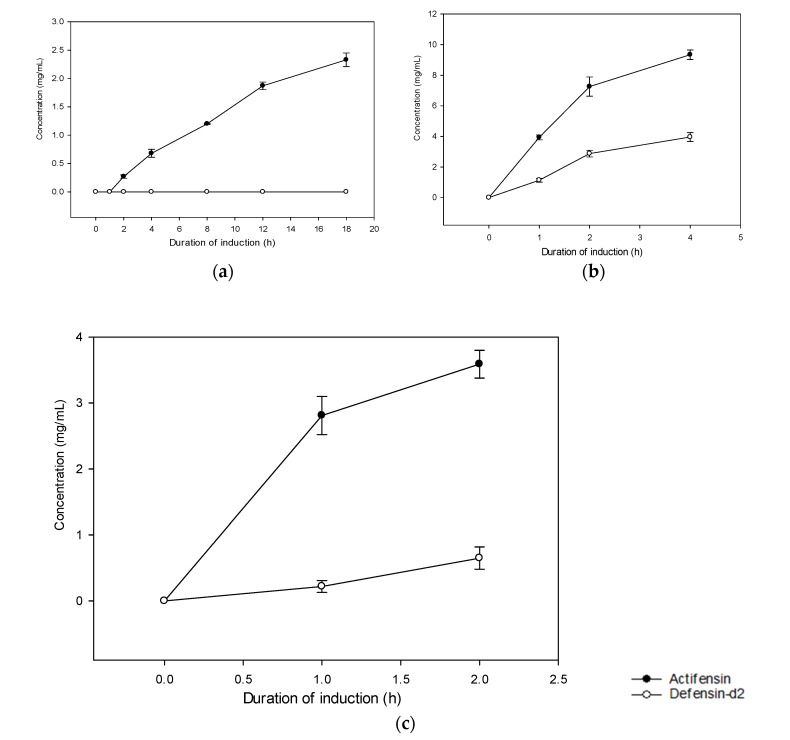
Time curves for the expressions of total soluble recombinant fusion proteins per induction condition: (**a**) expression at 15 °C for 18 h; (**b**) expression at 30 °C for 4 h; (**c**) expression at 37 °C for 2 h. Graph shows means and standard error means (error bars) of triplicate data per induction time.

**Figure 5:**
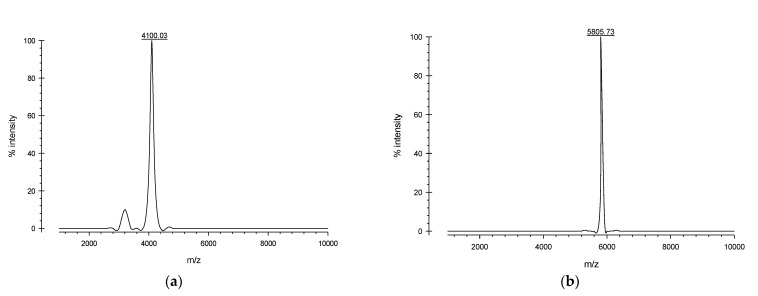
MALDI-TOF MS analysis and quantification of purified recombinant peptides: (**a**) MALDI-TOF MS analysis of purified recombinant actifensin showing a distinct peak corresponding to 4100.03 Da; (**b**) MALDI-TOF MS analysis of purified recombinant defensin-d2 showing a distinct peak corresponding to 5805.73 Da.

The SDS-PAGE analysis showed the expressions of soluble defensin–intein–CBD and actifensin–intein–CBD fusion proteins, which were estimated to be 40 kDa and 39 kDa, respectively (Figure 3a). Notably, defensin–intein–CBD was not expressed at induction for 18 h at 15 °C, but was expressed in soluble form at 30 °C and 37 °C. However, actifensin–intein–CBD was expressed at all induction conditions assessed. Both fusion proteins showed optimal expressions at induction with 0.4 mM of IPTG for 4 h at 30 °C (C2). The total protein concentrations, quantified by Bradford assay, were approximately 0.22–3.96 mg/mL and 0.27–9.34 mg/mL for defensin–intein–CBD and actifensin–intein–CBD, respectively, across the induction conditions (Figure 4). After the peptide cleavage and chitin affinity chromatography, the SDS-PAGE analysis showed the successful cleavage of the intein–CBD fusion partners, which resulted in purified recombinant peptides less than 6 kDa (Figure 3b,c). The MALDI-TOF MS analysis of the purified recombinant peptides showed single distinct peaks of 4.1 kDa and 5.8 kDa for actifensin and defensin-d2, respectively (Figure 5). The concentrations of the recovered purified recombinant peptides, quantified by Bradford assay, were approximately 0.16–0.98 mg/mL and 0.72–2.89 mg/mL for defensin-d2 and actifensin, respectively.

### 2.4. Antimicrobial Activity of the Recombinant Peptides

#### 2.4.1. Minimum Inhibitory Concentrations of Recombinant Peptides

The minimum inhibitory concentrations (MICs) of the recombinant peptides are presented in Table 2. The MICs of recombinant actifensin were 23, 45 and 1448 µg/mL against methicillin-resistant *S. aureus* (MRSA), *C. albicans* and *P. aeruginosa*, respectively. A broader range of antimicrobial activity was observed with recombinant defensin-d2: the MICs against *C. albicans*, *P. aeruginosa*, *K. pneumoniae* and *E. coli* were 7.5, 7.5, 30 and 30 µg/mL, respectively. It is noteworthy that both recombinant peptides exerted high potency against *C. albicans* and *P. aeruginosa* with MICs lesser than the standard antibiotics (ampicillin and nystatin). However, recombinant defensin-d2 exhibited higher potency against both organisms, as well as significant inhibitory activity against all Gram-negative bacteria. Generally, the recombinant peptides had lower MICs against the test organisms compared with the standard antibiotics, except vancomycin against MRSA. Both peptides exhibited bactericidal and fungicidal activity against *P. aeruginosa* and *C. albicans* (Table 2). Actifensin had an MBC of 724 µg/mL against *C. albicans* and 1448 µg/mL against *P. aeruginosa*, while defensin-d2 exerted cidal actions at 63, 123 and 246 µg/mL for *C. albicans*, *P. aeruginosa* and *E. coli*, respectively.

#### 2.4.2. Synergistic Activity of Recombinant Peptides

The determination of the antimicrobial synergy between actifensin and defensin against *C. albicans* and *P. aeruginosa* is presented in Table 3. Fractional inhibitory concentration index (FICI) values greater than 4 indicated that the interactions between both recombinant peptides are antagonistic. For both organisms, the MIC of defensin-d2 in combination with actifensin was 2^3^ higher than the MIC alone. While the MIC of actifensin against *P. aeruginosa* remained unchanged in combination with defensin-d2, there was a 2^3^ increase in the MIC against *C. albicans*.

#### 2.4.3. Inhibitory Kinetics of Recombinant Actifensin and Defensin-d2

**Figure 6:**
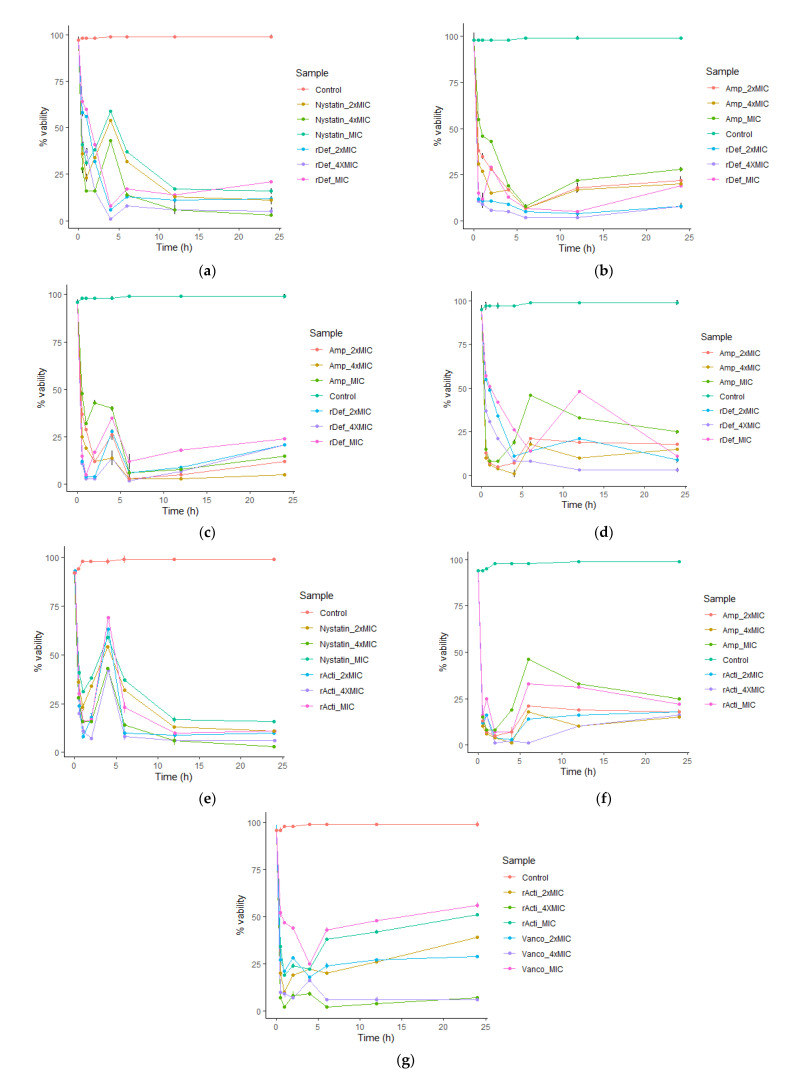
Inhibition kinetics of recombinant peptides: (**a**) *C. albicans* vs. defensin-d2; (**b**) *P. aeruginosa* vs. defensin-d2; (**c**) *E. coli* vs. defensin-d2; (**d**) *K. pneumoniae* vs. defensin-d2; (**e**) *C. albicans* vs. actifensin; (**f**) *P. aeruginosa* vs. actifensin; (**g**) MRSA vs. actifensin. Graph shows means and standard error means (as error bars) of triplicate data per time point.

The inhibition kinetics of the recombinant peptides against the test organisms are presented in Figure 6. The killing kinetics of the recombinant defensin against *C. albicans* was concentration dependent. The onset of fungicidal activity was within the 1st hour of exposure, and it remained consistent until the 24th hour. All concentrations (7.5–30 µg/mL) of defensin-d2 reduced the cell viability of *C. albicans* to below 20% (Figure 6a). For *P. aeruginosa*, bactericidal activity started within the first 6 h, which is similar to ampicillin. While a decrease in cidal action was observed in ampicillin from the 6th hour, a slight increase in the cell viability was only observed at the 12th hour in the MIC and 2× MIC of defensin-d2. Notwithstanding, the MIC of defensin (7.5 µg/mL) reduced the viability to about 10% after 24 h (Figure 6b). Against *E. coli*, defensin-d2 exhibited the onset of bactericidal activity within the 1st hour of exposure, and consistently reduced the cell viability by >75% throughout the 24 h observation, unlike ampicillin (Figure 6c). For *K. pneumoniae,* there was a bactericidal action within the first 4 h, but it reduced steadily from the 6th hour up to the 24th across all concentrations of defensin-d2 and ampicillin (Figure 6d).

The recombinant actifensin also revealed a concentration-dependent fungicidal action against *C. albicans*, with similar kinetics to nystatin. Although there was an initial contact inhibitory effect caused by actifensin in the first 2 h, significant fungicidal activity was observed after the first 4 h, with a steady decline in the percentage viability of *C. albicans* cells up to 24 h. Specifically, the MIC of actifensin (23 µg/mL) reduced the percentage viability to below 10% after 24 h (Figure 6e). Actifensin also inhibited *P. aeruginosa* in a pattern similar to that of ampicillin, with a decrease in the cell viability below 25% after 6 h in all concentrations. Similar to the observations for *C. albicans,* an early onset of bactericidal action in the first 4 h at all concentrations was also observed against *P. aeruginosa* (Figure 6f). While actifensin drastically reduced the cell viability of *C. albicans* and *P. aeruginosa* at all concentrations, a contrasting kinetic was observed for MRSA, as only the 4× MIC (92 µg/mL) exerted notable bactericidal action up to 24 h (Figure 6g).

#### 2.4.4. Resistance Potential of Test Organisms to Recombinant Actifensin and Defensin-d2

**Figure 7:**
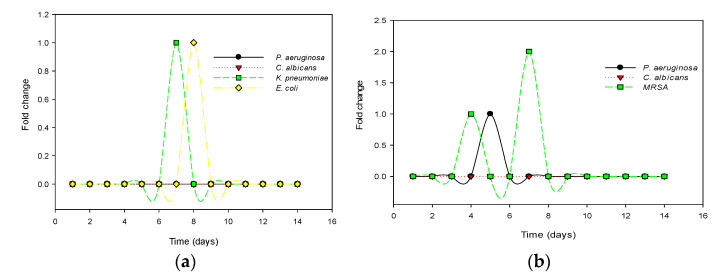
Resistance potentials of the test organisms against the recombinant peptides: (**a**) fold change in MIC of recombinant defensin-d22; (**b**) fold change in MIC of recombinant actifensin.

The resistance potentials of the sensitive test organisms to recombinant defensin-d2 and actifensin are presented in Figure 7. Out of the four sensitive organisms subjected to prolonged exposure, and serial passages of defensin-d2 to induce selective pressure, *C. albicans* and *P. aeruginosa* showed no fold changes in the MICs. *E. coli* and *K. pneumoniae* both showed 2^1^-fold changes in the MICs, from 30 to 60 µg/mL, which remained unchanged for the rest of the assay (Figure 7a). For actifensin, *C. albicans* showed no resistance, despite repeated passages. However, *P. aeruginosa* showed a fold change in the MIC from 1448 to 2896 µg/mL, while the MIC of MRSA shifted from 23 to 181 µg/mL (Figure 7b). However, no MIC changes were observed after day 10 for all the organisms, which indicated that there was no resistance potential of the test organisms beyond 2–4 × the MICs of both recombinant peptides.

### 2.5. Hemolytic Activity of Recombinant Actifensin and Defensin-d2

**Figure 8:**
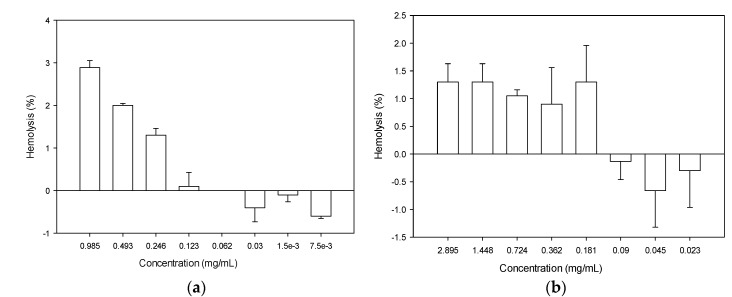
Hemolytic activity of (**a**) recombinant defensin-d2 and (**b**) recombinant actifensin. Graph shows means and standard error means (as error bars) of triplicate data per concentration.

To determine the toxicity of the recombinant peptides, the hemolytic activity was determined using mouse erythrocytes, and the results are presented in Figure 8. For defensin-d2, the hemolytic activity increased directly with the increase in the concentration. The maximum hemolysis of 2.89% was observed at a concentration of 985 µg/mL. It is worth noting that none of the MICs up to 8× the MICs determined for the test organisms showed any potential for hemolysis (Figure 8b). On the other hand, the hemolytic activity of actifensin was not concentration dependent. However, the hemolysis was less than 1.5% in all concentrations. Moreover, concentrations less than 181 µg/mL showed no potential for hemolysis.

### 2.6. Biofilm-Inhibition Potential of Recombinant Actifensin and Defensin-d2

**Figure 9:**
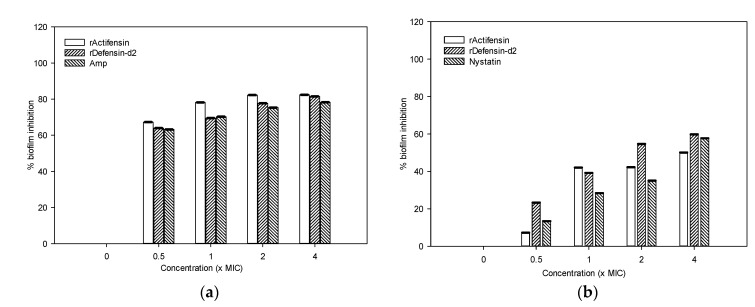
Inhibitory activity of the recombinant peptides on biofilm formation by (**a**) *Pseudomonas aeruginosa* and (**b**) *Candida albicans*. Graphs show means and standard error means (as error bars) of triplicate data per concentration.

As shown in Figure 9, defensin-d2 and actifensin inhibited biofilm formation in MDR *P. aeruginosa* and *C. albicans* in a concentration-dependent manner. After treatment with 0.5–4× the MIC of defensin-d2 for 24 h, the percentages of biofilm mass in *P. aeruginosa* decreased by over 70%, which is comparable to the effect of ampicillin. Both recombinant peptides exhibited lesser biofilm inhibition against *C. albicans*; the percentage of biofilm mass decreased by ~40% when the concentration of peptides was up to 4× the MIC. This suggests that defensin-d2 and actifensin can effectively inhibit biofilm formation in MDR *P. aeruginosa* and *C. albicans* at an early stage.

### 2.7. Peptide–Ligand Interactions of Recombinant Actifensin and Defensin-d2

**Figure 10:**
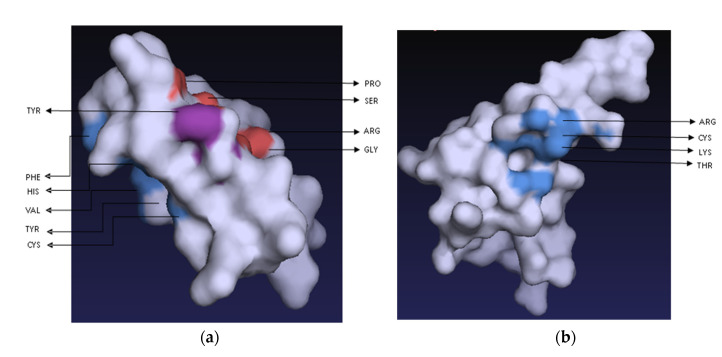
Binding-pocket sites and interacting residues on (**a**) actifensin and (**b**) defensin-d2 structures predicted using PrankWeb.

Both peptides showed almost identical ligand-affinity propensities with the 14 ligands screened: ACT, PO_4_, EDO, PA8, PGE, MRD, P10, 44E, SO_4_, GOL, CL, FLC, PEG and 13C (Table S1). Both peptides showed high affinities to ACT (acetate ion), PO_4_ (phosphate ion), EDO (1,2-Ethanediol) and MRD (S(4r)-2-Methylpentane-2,4-Diol), but no affinity to PA8 (1,2-Dioctanoyl-Sn-Glycero-3-Phosphate) and 44E ((2R)-3-(phosphonooxy) propane-1,2-diyl dihexanoate). Two amino acid residues, arginine (R) and cysteine (C), were found to be common in the binding pockets of both peptides (Figure 10).

### 2.8. Membrane Permeability by Recombinant Defensin-d2 and Actifensin

#### 2.8.1. Outer-Membrane Permeability by Recombinant Defensin-d2 and Actifensin

**Figure 11:**
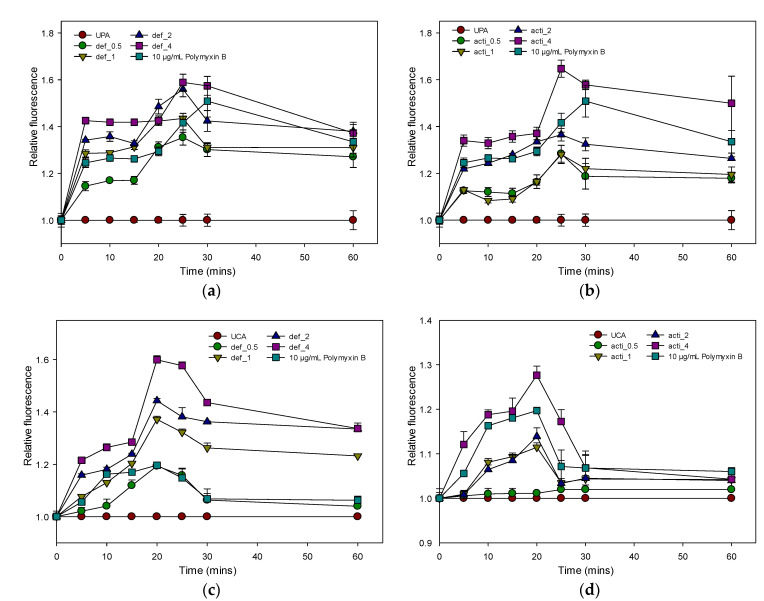
Relative fluorescence of NPN uptake to assess outer-membrane permeability of recombinant defensin-d2 and actifensin against *P. aeruginosa* and *C. albicans*. (**a**) defensin-treated *P. aeruginosa* (**b**) actifensin-treated *P. aeruginosa* (**c**) defensin-treated *C. albicans* (**d**) actifensin-treated *C. albicans*. Untreated samples were used for normalization. UPA—untreated *P. aeruginosa*; UCA—untreated *C. albicans*; def—defensin-d2; acti—actifensin. Graphs show means and standard error means (as error bars) of triplicate data per treatment.

The outer-membrane permeabilizations of MDR *P. aeruginosa* and *C. albicans* were determined by using the fluorescent dye 1-N-phenylnapthylamine (NPN) uptake assay. As shown in Figure 11, defensin-d2 and actifensin rapidly permeabilized the outer membranes of *P. aeruginosa* and *C. albicans* in a concentration-dependent manner, as observed by the increase in the NPN fluorescence. The peptides were able to permeabilize the outer membranes of both organisms, and especially *P. aeruginosa*, even at 0.5× the MICs for both peptides. The increase in the fluorescence observed at 2× and 4× the MICs of both recombinant peptides was higher than that of the 10 µg/mL polymyxin B.

#### 2.8.2. Plasma-Membrane Permeability by Recombinant Defensin-d2 and Actifensin

**Figure 12:**
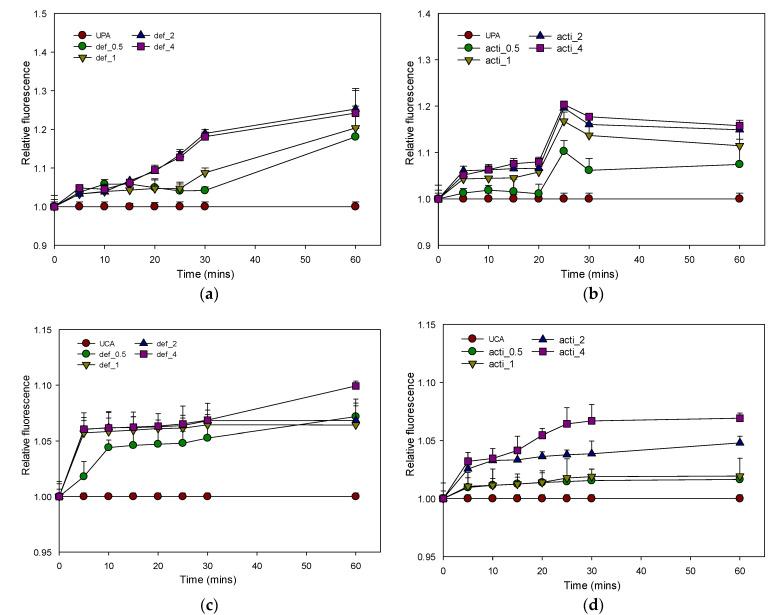
Relative fluorescence of PI uptake to assess plasma-membrane permeability of recombinant defensin-d2 and actifensin against *P. aeruginosa* and *C. albicans*. (**a**) defensin-treated *P. aeruginosa* (**b**) actifensin-treated *P. aeruginosa* (**c**) defensin-treated *C. albicans* (**d**) actifensin-treated *C. albicans*. Untreated samples were used for normalization. UPA—untreated *P. aeruginosa*; UCA—untreated *C. albicans*; def—defensin-d2; acti—actifensin. Graphs show means and standard error means (as error bars) of triplicate data per treatment.

Following the exposure of MDR *P. aeruginosa* and *C. albicans* to 0.5–4× MICs of recombinant defensin-d2 and actifensin, plasma permeabilization was observed with increased propidium iodide (PI) fluorescence. In *P. aeruginosa*, both peptides significantly permeabilized the plasma membrane within 30 min, while strongly evident permeabilization was observed within 5 min in *C. albicans*, and especially with the defensin-d2 treatment (Figure 12).

#### 2.8.3. Inner-Membrane Depolarization by Recombinant Defensin-d2 and Actifensin

**Figure 13:**
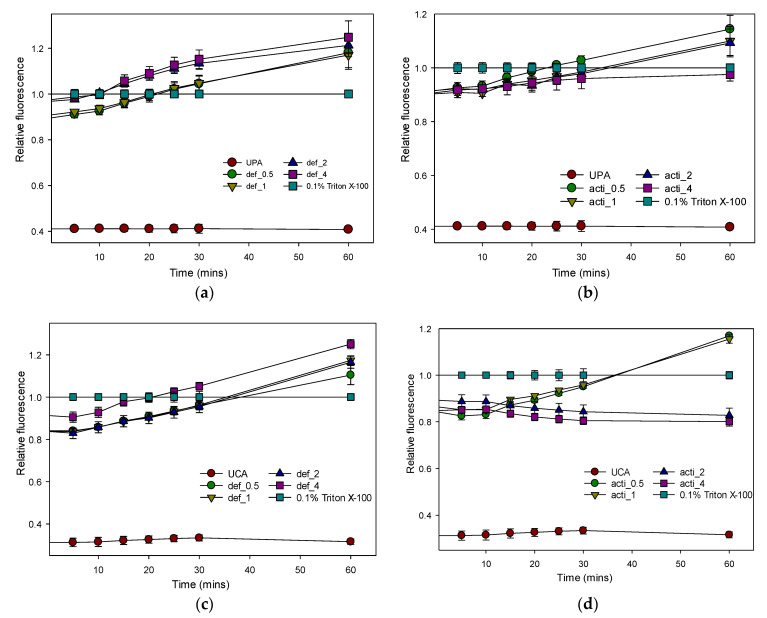
Relative fluorescence of 3-DiSc (5) uptake to assess inner-membrane depolarization of recombinant defensin-d2 and actifensin against *P. aeruginosa* and *C. albicans*. (**a**) defensin-treated *P. aeruginosa* (**b**) actifensin-treated *P. aeruginosa* (**c**) defensin-treated *C. albicans* (**d**) actifensin-treated *C. albicans*. The positive control, 0.1% (*v*/*v*) Triton-X 100, was used for normalization of fluorescence. UPA—untreated *P. aeruginosa*; UCA—untreated *C. albicans*; def—defensin-d2; acti—actifensin. Graphs show means and standard error means (as error bars) of triplicate data per treatment.

The membrane potential-sensitive dye 3,3 -Dipropylthiadicarbocyanine iodide (diSC3(5)) was used to evaluate the depolarization of the recombinant defensin-d2 and actifensin on the cytoplasmic membranes of MDR *P. aeruginosa* and *C. albicans*. The results showed that both peptides induced a concentration-dependent increase in diSC3(5) fluorescence, which indicated cytoplasmic membrane depolarization (Figure 13). Recombinant defensin-d2 and actifensin were more effective and rapid at permeabilizing the inner membrane at concentrations of 2*×* and 4× their MICs. A significant increase in the fluorescence intensity was observed within 5 min of the exposure of both organisms to the recombinant peptides.

#### 2.8.4. Effect of Recombinant Defensin-d2 and Actifensin on ROS Production

**Figure 14:**
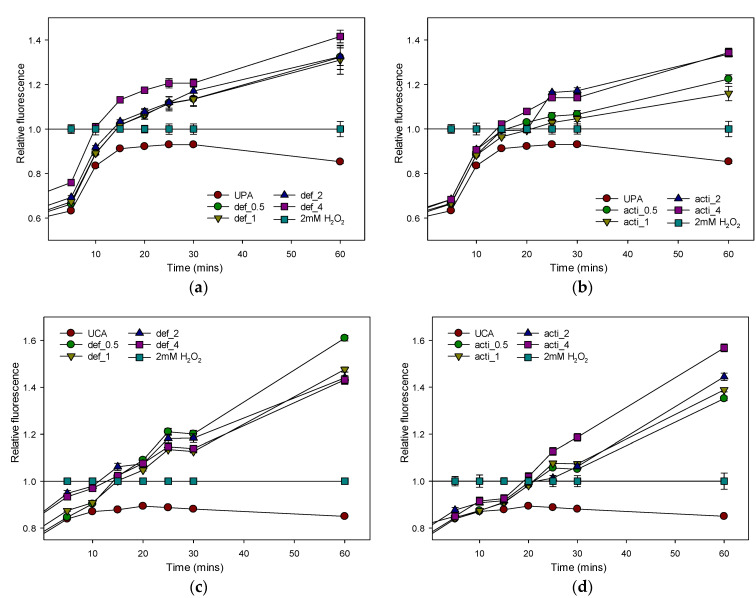
Relative fluorescence of H_2_DCFDA for assessing ROS production in *P. aeruginosa* and *C. albicans* treated with recombinant defensin-d2 and actifensin. (**a**) defensin-treated *P. aeruginosa* (**b**) actifensin-treated *P. aeruginosa* (**c**) defensin-treated *C. albicans* (**d**) actifensin-treated *C. albicans*. The positive control, 2mM H_2_O_2_, was used for normalization of fluorescence. UPA—untreated *P. aeruginosa*; UCA—untreated *C. albicans*; def—defensin-d2; acti—actifensin. Graphs show means and standard error means (as error bars) of triplicate data per treatment.

The cell-permeant dye 2′,7′-dichlorodihydrofluorescein diacetate (H_2_DCFDA) was used to assess the reactive-oxygen-species (ROS) production in the test organisms. As shown in Figure 14, increased ROS production in MDR P. aeruginosa and C. albicans was induced by exposure to 0.5–4× MICs of recombinant defensin-d2 and actifensin. The ROS production in both organisms increased within 10 min of the peptide treatment, even at 0.5× the MICs, which indicated that both peptides were significantly capable of enhancing the ROS production in both test organisms (Figure 14).

## 3. Discussion

### 3.1. Bioinformatics Analysis of the Peptides

Antimicrobial peptides (AMPs), and especially defensins and defensin-like classes, hold great prospects as effective antimicrobials against MDR pathogens. Studies have shown that the presence of highly conserved cysteine residues and disulfide bonds in defensins and defensin-like AMPs provide high stability, which is a crucial feature that is needed for the efficacy of antimicrobials [17,18]. The in silico characterization of defensin-d2 and actifensin that was conducted in this study affirms previous reports on the estimated molecular weights and cysteine-rich features of the peptides [8,10]. In defensin-d2, the eight cysteine residues in the peptide sequence were predicted to form C1–C8, C2–C5, C3–C6 and C4–C7 disulfide bonds, while, in actifensin, the cysteine residues formed three disulfide bonds at C1–C4, C2–C3 and C5–C6. The disulfide connectivity patterns of both peptides, based on the pairing of the cysteine residues, indicated that they have the αβ architectural conformation [19,20]. The cysteine-stabilized αβ motif is a peculiar feature of defensins from plants, mussels, insects, and fungi [17], which thus makes actifensin derived from *Actinomyces ruminicola* a unique bacteriocin, with a high similarity to plant and fungal defensins, as previously reported [10].

The in silico characterization of the peptides further showed that both peptides have an overall cationic net charge, which could be attributed to the higher composition of positively charged residues (arginine and lysine) in both peptides. We also determined the hydropathy of the peptides by calculating the GRAVY score. The hydropathy of peptides is crucial to their biological activities, bioavailability distribution and molecular interactions, which determine the mechanism of action [21]. In principle, the more positive the GRAVY score, the more hydrophobic (or membranous) the peptide is [22]. The negative GRAVY scores obtained for both peptides measured by Kyte–Doolittle [23] suggest that the two peptides are globular, hydrophobic in nature and have a high tendency of efficient solubility. This prediction correlates with the alternating hydrophobic and hydrophilic properties of the amino acid residues of both peptides, which further affirm the amphipathic nature conferred on them by the αβ structural conformation [17].

Moreover, the predictions of both peptides as globular indicates that they are functional rather than structural, and, as such, sensitive to environmental changes, such as temperature and pH [24]. In line with this, the instability index (II), which estimates the stability of proteins experimentally [25], suggests that actifensin is more stable than defensin-d2, experimentally. This may suggest that, despite the similarity of both peptides, defensin-d2 contains certain dipeptides that significantly reduce its overall stability. The dipeptide composition of peptides was reported to determine its overall stability because certain dipeptides are more prone to degradation and in vivo absorption [26]. To further examine the stability of the peptides in this study, the aliphatic index, which is a value that is regarded as a measure of the thermostability of globular proteins, was determined. Its values also indicated that actifensin (47.30) is more likely to remain stable over a range of temperatures than defensin-d2 (24.42). In principle, the higher the aliphatic index, the more thermally stable the protein over a wide range of temperatures [27,28].

### 3.2. Generation of Recombinant Plasmids and Protein Expression

The use of a restriction- and ligation-independent cloning strategy in this study generated recombinant plasmids with the correct orientation of the target genes, without the incorporation of unwanted nucleotides that may alter the desired peptides produced. Furthermore, we demonstrated the successful use of MEGAWHOP PCR as a cloning strategy for small fragments (<300 bp) of target genes. MEGAWHOP uses the DNA fragment to be cloned as a set of complementary primers that replace a homologous region in a template vector through a megaprimer PCR of the whole plasmid [29,30]. A modification to the technique made in this study was the linearization of the plasmid by PCR to further enhance homologous priming. Another advantage of this strategy is that the gel purification of the recombinant plasmids is not required because the PCR product can be used directly for transformation, as used in this study. This study also utilized an *E. coli* system for the heterologous production of soluble forms of the peptides due to its rapid multiplication, ease of upscaling and optimization and low costs compared with other expression systems [31]. Moreover, to enhance the success of the expressions of these small peptides in *E. coli*, we expressed them as fusion proteins, with Mxe GyrA intein and chitin-binding domain (CBD) as fusion partners. Our study therefore reiterates previous reports that state that the use of fusion partners increased the success of the recombinant expression of AMPs in *E. coli* by enhancing the solubility, repressing the toxicity against the host cell and improving the purification process [16]. In our study, the temperature and duration of induction were factors that greatly influenced the expressions and yields of the recombinant peptides. The yields of the recovered purified recombinant peptides were 31% (2.89 mg/mL) and 25% (0.98 mg/mL) of the total fusion proteins expressed for actifensin and defensin-d2, respectively. Previous studies reported recoveries of 0.0025–0.05 mg/mL of plant defensins [5,32,33] and 0.18–1.85 mg/mL of bacteriocins [34,35,36] in *E. coli*. Our study has therefore demonstrated a high-yield heterologous production of plant- and actinomyces-derived defensin and defensin-like peptides, with considerably high purity, in an *E. coli* system.

### 3.3. Antimicrobial Activity of Purified Recombinant Peptides

A critical aspect to the recombinant production of antimicrobial molecules of different origins in *E. coli* is the possibility of a loss of biological activity or potency due to misfolding [31]. In this study, the purified recombinant peptides showed notable inhibitory potentials against multidrug-resistant strains of *Pseudomonas aeruginosa*, *Klebsiella pneumoniae*, *Candida albicans* and methicillin-resistant *Staphylococcus aureus* (MRSA), as well as *Escherichia coli*. These pathogens are considered a critical priority by the WHO for antibiotics research and development because of their propensity to increase the fatality and mortality rates of infections [37]. Recombinant actifensin and defensin-d2 showed different antimicrobial patterns, but they both had inhibitory effects against *P. aeruginosa* ATCC 27853 and *C. albicans* ATCC 64124. Specifically, recombinant actifensin was active against MRSA (*S. aureus* ATCC 43300) at a low MIC, but not against *E. coli* ATCC 25922 and *K. pneumoniae* ATCC 700603. This result agrees with the reports of Sugrue et al. [10], which state that actifensin did not inhibit *E. coli*, but showed strong inhibition against MRSA. Although the majority of studies have shown the narrow-spectrum activity of bacteriocins, the recombinant actifensin in our study showed antimicrobial activity against both Gram-positive and -negative bacteria, as well as yeast. Yu et al. [35] also reported the broad-spectrum activity of a recombinant LacAB bacteriocin from *Lactobacillus casei*. Additionally, the inhibitory activity of bacteriocins against *Candida* and Gram-negative bacteria, including *E. coli* ATCC 8739, *Shigella* sp., *Salmonella* sp. and *Pseudomonas* sp., have been reported [36,38,39].

Recombinant defensin-d2 showed antibacterial activity against all the Gram-negative bacteria tested, but not against MRSA. In addition, defensin-d2 exhibited remarkable antifungal activity against *C. albicans* at an MIC lower than that of nystatin. Crude and chemically synthesized defensin-d2 has been shown to possess antimicrobial activity against *P. aeruginosa* PAO1, *E. coli*, *Serratia marcescens*, *Enterobacter aerogenes*, *Xanthomonas alfalfa*, *Clavibacter michiganensis*, *Ralstonia solanacearum* and *Fusarium* sp. [8,9,40]. These previous studies, similar to most studies on plant defensins, have extensively focused on the antimicrobial activity of defensin-d2 against plant pathogens, and specifically its antifungal activity. However, in this study, we determined that recombinant defensin-d2 possess strong antibacterial and antifungal activities against multidrug-resistant human pathogens. Comparatively, recombinant defensin-d2 showed higher and broader potency with lower MICs than recombinant actifensin. While the presence of the cysteine-stabilized αβ structural conformation has been postulated as the determinant of antimicrobial activity in defensins and defensin-like peptides [17,20], the results of our study further reiterate the importance of the entire peptide composition on the antimicrobial activity and specificity of AMPs.

The inhibitory kinetics of the recombinant peptides showed that the MICs of actifensin (23 µg/mL) and defensin-d2 (7.5 µg/mL) exhibited fungicidal actions that reduced the cell viability of *C. albicans* by over 90% within 24 h, which was comparable to the effects of 4 × the MIC (129 µg/mL) of nystatin. However, the effects of the recombinant peptides against the bacteria tested were quite different. On the one hand, recombinant actifensin showed a more bacteriostatic action against *P. aeruginosa* and MRSA. On the other hand, recombinant defensin-d2 exerted a consistent bactericidal action against *P. aeruginosa* and *E. coli*, but not *K. pneumoniae*. Although the clinical reliance of classifying antimicrobial agents as static or cidal is questioned due to its dependence on the drug concentration and pathogen, this pharmacological classification is still successfully used to discriminate the antimicrobial actions of potential drug candidates [41]. Overall, the antimicrobial activity of the recombinant peptides compared was similar or better than the standard antibiotics used, except for MRSA.

We also determined that the antimicrobial synergy of both recombinant peptides against *C. albicans* and *P. aeruginosa* was antagonistic. We postulate that this phenomenon may be attributed to the utilization of a similar channel of interaction by the recombinant peptides against the sensitive organisms, which thereby causes a competitive antagonism. One of the causes of antagonism in drug interactions is the use of the same receptor or ligand by two individually efficacious drugs, which results in competitive (causing an increase in the drug concentration required, as seen in this study) or noncompetitive (irreversible; the increase in the drug concentration is inconsequential) antagonism [42,43]. Our postulation was further corroborated by the almost identical peptide–ligand-interaction profiles of both peptides, which indicates that both peptides interact at highly similar affinities with the same ligands and have similar residues on the surfaces of their binding pockets. We also determined that there was no induction of resistance in the sensitive organisms to the recombinant peptides over repeated exposure for 20 consecutive days. This further supports the reports that state that AMPs are less prone to resistance by pathogens [44], which is possibly due to their ability to exert multiple mechanisms of action concurrently [15].

This study also investigated the ability of recombinant defensin-d2 and actifensin to inhibit biofilm formation in *C. albicans* and *P. aeruginosa*. Biofilm formation contributes to virulence and inherent resistance in pathogens because of their high tolerance to environmental stress [45,46], and to the extracellular polysaccharide matrix that surrounds biofilms, which prevents the penetration of antimicrobials [44]. Specifically, *C. albicans* and *P. aeruginosa* are predominant species in biofilm-associated infections and the colonization of medical devices, with the potential to cause systemic bloodstream, tissue and organ infections [46,47]. As a result, biofilm-associated infections pose a critical health risk that demands the urgent development of antibiofilm compounds. Notably, both recombinant peptides significantly inhibited the formation of biofilms of *C. albicans* and *P. aeruginosa*, even at concentrations less than their MICs.

### 3.4. Toxicity of the Recombinant Peptides

A major limitation to the clinical use of antimicrobial peptides is their toxicity to mammalian cells [48]. Both recombinant actifensin and defensin-d2 showed low hemolysis below 3% at the maximum concentrations of both peptides. The highest hemolysis of 2.89% was observed at 985 µg/mL, which is 128 times higher than the MICs for *C. albicans* and *P. aeruginosa*, and 32 times higher than the MICs for *E. coli* and *K. pneumoniae*. Similarly, the maximum hemolysis of 1.3% was observed at the highest concentration (2895 µg/mL) of actifensin, which is about 128, 64 and 2 times higher than the MICs of *C. albicans*, MRSA and *P. aeruginosa*, respectively. Overall, both recombinant peptides have low toxicity.

### 3.5. Cell Permeability of Recombinant Actifensin and Defensin-d2

In this study, we established that membrane permeability is a mechanism of action of recombinant defensin-d2 and actifensin against MDR *C. albicans* and *P. aeruginosa.* An increase in the fluorescence of NPN and PI, which are dyes that are normally excluded by intact cell membranes [49], seen in the test organisms treated with the recombinant peptides indicated the significant disruption of the outer and plasma membranes of the organisms within 1 h. Moreover, the cytoplasmic-membrane depolarization potential of the recombinant peptides was assayed by using the membrane potential-sensitive dye diSC_3_(5), which concentrates in the intact cytoplasmic membrane under the influence of the membrane potential, which results in a self-quenching of the fluorescence [50]. Both peptides caused an increase in diSC_3_(5) fluorescence, even at subinhibitory concentrations, which indicates that the accumulated dye was released due to pore formation or the disruption of the cytoplasmic membrane [50,51,52]. We also assessed the induction of the production of reactive oxygen species (ROS) by recombinant defensin-d2 and actifensin in MDR *C. albicans* and *P. aeruginosa*. Both peptides increased the ROS production in both organisms by >30% within 15 min of exposure. This observation is consistent with the membrane damage seen in the membrane-permeability assays because increased ROS production is a key indicator of oxidative stress in cells, which is usually associated with cellular damage [53]. Excessive ROS production can affect lipids, protein and nucleic acid metabolism, which leads to cell death [54]; thus, we established that the induction of ROS production also plays a key role in the mechanism of action of recombinant defensin-d2 and actifensin against MDR *C. albicans* and *P. aeruginosa*. Our study also supports reports that state that cationic AMPs have a membrane-targeted mechanism of action against bacteria and fungi that is mediated by electrostatic interactions with the anionic cell membranes of the microbes [55,56].

## 4. Materials and Methods

### 4.1. Test Organisms

Cultures of *Pseudomonas aeruginosa* ATCC 27853, *Staphylococcus aureus* ATCC 43300 (MRSA), *Escherichia coli* ATCC 25922, *Klebsiella pneumoniae* ATCC 700603 and *Candida albicans* ATCC 64124 were purchased from the American Type Culture Collection (ATCC). The propagation conditions for the isolates were followed as recommended by the ATCC, and isolates were preserved at 4 °C.

### 4.2. Bioinformatics Analysis

The amino acid sequence of spinach defensin-d2 was retrieved from UniprotKB, with the accession number P18571, while the actifensin sequence was retrieved from Sugrue et al. [10]. The isoelectric points (pIs) and molecular weights of the peptides were computed using the Expasy pI/Mw tool [57] (https://web.expasy.org/compute_pi, accessed on 6 June 2022). Signal peptides in the peptides were predicted using SignalP 5.0 [58] (https://services.healthtech.dtu.dk/service.php?SignalP-5.0, accessed on 6 June 2022). The hydropathy of the peptides was determined by calculating the grand average of hydropathy (GRAVY) and average hydrophilicity scores (http://www.gravy-calculator.de, accessed on 6 June 2022). The net charge at physiological pH, aliphatic index (AI) and instability index (II) of the peptides were deduced using the ProtParam tool [57] (https://web.expasy.org/protparam, accessed on 6 June 2022). The positions of the disulfide bonds were also predicted using the DiANNA 1.1 web server [59] (http://clavius.bc.edu/~clotelab/DiANNA/, accessed on 6 June 2022).

### 4.3. Generation of Gene Constructs

Since the actual DNA sequences of the genes encoding defensin-d2 and actifensin are unknown, the amino acid sequences available were reverse-translated using EMBOSS Backtranseq [60] (https://www.ebi.ac.uk/Tools/st/emboss_backtranseq, accessed on 6 June 2022). For the reverse-translation of defensin-d2, the *Spinacia oleracea* codon was used, while the *Streptomyces coelicolor* A3 codon was used to reverse-translate actifensin. The nucleotide sequences were synthesized as gene constructs cloned into pJET 1.2 by Genscript (Hongkong, China).

#### PCR Amplification of Plasmid and Inserts

The pTXB1 plasmid (New England Biolabs, Ipswich, MA, USA) was linearized by PCR at the multiple cloning sites (MCSs). In parallel, the fragments encoding actifensin and defensin-d2 were amplified from pJET 1.2, carrying each of the synthetic genes. The fragments were amplified with primers containing homologous sequences of 15 bp upstream and downstream of the MCS of the pTXB1 vector. Moreover, the primers were designed to allow an in-frame insertion of the fragments with the fusion proteins, Mxe GyrA intein and chitin-binding domain (CBD), on pTXB1. The primers used for plasmid linearization and fragment amplification are listed in Table 4. The fragments encoding actifensin and defensin-d2 were amplified from 20 ng of pJET 1.2 in a 50 µL reaction using Phusion High-Fidelity DNA Polymerase (New England Biolabs, Ipswich, MA, USA). The PCR amplicons were purified using the ISOLATE II PCR and Gel kit (Bioline, London, UK), and were quantified using a Nanodrop spectrophotometer (Jenway Genova Nano, London, UK), followed by the confirmation of a successful recovery by resolving 10 µL of the purified products on 1.5% (*w*/*v*) RedSafe ™-stained agarose gel at 80 V for 40 min.

### 4.4. Gene Cloning

**Figure 15:**
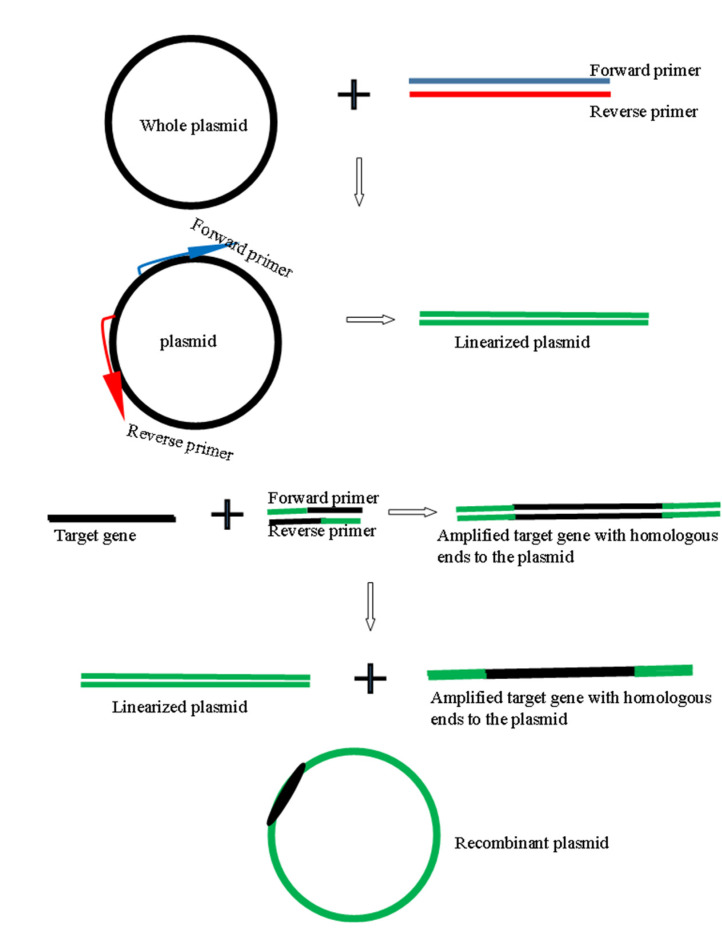
Schematic illustration of the cloning strategy employed.

In the MEGAWHOP cloning strategy [29,30], the amplified PCR products of the target genes were used as megaprimers in a PCR (illustrated in Figure 15) with 20 ng of pTXB1 using Phusion High-Fidelity DNA Polymerase (New England Biolabs, Ipswich, MA, USA), according to the manufacturer’s instructions. The PCR amplicons were incubated with 10 U of DpnI (New England Biolabs, Ipswich, MA, USA) for 1 h to digest the parental plasmid. Afterwards, the recombinant pTXB1 plasmids—pTXB1-actifensin and pTXB1-defensin-d2—were purified using the ISOLATE II PCR and Gel kit (Bioline, London, UK), and resolved on 0.8% RedSafe™-stained agarose gel at 80 V for 40 min. The recombinant plasmids were also digested with restriction enzymes: pTXB1-actifensin was digested with *HindIII* and *BamHI*, while pTXB1-defensin-d2 was digested with *EcoRV* and *BamHI*. The digested fragments were also resolved on 0.8% (*w*/*v*) RedSafe™-stained agarose gel at 80 V for 40 min.

#### 4.4.1. Transformation of *E. coli* and Clone Verification

Five microliters of recombinant plasmids containing actifensin and dfensin-d2 were separately transformed into 50 µL of competent *E. coli* Shuffle T7 cells (New England Biolabs, Ipswich, MA, USA) by heat shock [61] at 42 °C for 30 s. The cells were incubated in 950 µL of modified SOC medium at 37 °C for 1 h. A 1:10 dilution of the cells was made in the modified SOC medium, and was subsequently plated on pre-warmed LB agar (Oxoid, Basingstoke, UK), supplemented with 100 µg/mL ampicillin, and then incubated at 30 °C for 24 h.

#### 4.4.2. Colony PCR and Plasmid Sequencing

Colony PCR was used to verify positive clones using vector- and gene-specific primers, as described by Packeiser et al. [62], with modifications. Discrete colonies were carefully picked from the antibiotic selection plates using a sterile micropipette tip. Part of the inoculum was first patched on an ampicillin-supplemented LB agar plate for replication, then the tip was transferred into 15 µL of the lysis buffer (Tris-EDTA pH 8.0 with 0.1% (*v*/*v*) Triton X-100). The colony suspensions were heated at 95 °C for 5 min, then centrifuged at 10,000 rpm for 30 s. A volume of 2 µL from the supernatant was used in a 25 µL PCR reaction using MyTaq™ DNA Polymerase (Bioline, London, UK). Vector- and gene-specific primers (Table 4) were used to screen colonies by PCR. 

The PCR-positive colonies were grown in 10 mL LB broth (Oxoid, Basingstoke, UK), supplemented with 100 µg/mL ampicillin, by overnight incubation at 37 °C. The alkaline lysis method was used for plasmid isolation using the GeneJet Plasmid Mini-prep kit (Thermo Scientific, Waltham, MA, USA). Purified plasmids were quantified using a Nanodrop spectrophotometer (Jenway Genova Nano, London, UK), and were resolved on 1% (*w*/*v*) RedSafe ™-stained agarose gel at 80 V for 40 min.

The recombinant plasmids were sequenced by the Sanger method using the T7 universal primer to confirm the orientation of the insert. Trace files were analyzed with Snapgene v 1.1.3, and pairwise alignments of the sequences of the gene constructs with the recombinant plasmids were performed to confirm the presence of inserts, and whether they are in-frame.

### 4.5. Expression and Optimization of Recombinant Defensin-d2 and Actifensin

Freshly grown colonies of positive clones were inoculated in 10 mL of LB broth (Oxoid, Basingstoke, UK), containing 100 µg/mL of ampicillin, at 37 °C for 24 h, with gentle agitation at 150 rpm. Then, 2 mL of the bacterial suspension was inoculated into 200 mL of Terrific broth, modified (Sigma Aldrich, Schnelldorf, Germany), containing 100 µg/mL of ampicillin, followed by incubation at 37 °C, with shaking at 150 rpm until the OD_600_ reached >0.5. An aliquot of 5 mL of *E. coli* culture was taken as an uninduced negative-control sample. Protein expression was induced using a final concentration of 0.4 mM of IPTG. The induced samples were incubated at 15 °C for 18 h (C1), 30 °C for 4 h (C2) and 37 °C for 2 h (C3) to determine the optimal conditions for protein expression. Samples were also taken at defined time points to monitor the rate of protein expression, and the cells were harvested by centrifugation at 5000× *g* for 20 min at 4 °C. The supernatant was discarded, and the cell pellet was weighed to determine the wet mass of the cells, prior to resuspension in 20 mL ice-cold column buffer (Tris-HCl pH: 8.0; 500 mM NaCl). The freeze–thaw method was used for *E. coli* lysis by subjecting cell pellets to 8–10 repeated cycles of freezing at −80 °C and thawing at 37 °C in a Precision GP 02 water bath (Thermo Fisher Scientific, Waltham, MA, USA). These cycles continued until the cell suspension became viscous, which indicated cell lysis. The suspension was centrifuged at 5000× *g* for 20 min at 4 °C. The clarified lysate was collected into precooled tubes and stored at −20 °C until use. The expression of recombinant defensind2–intein–CBD and actifensin–intein–CBD fusion proteins were detected by 4–20% tricine SDS-PAGE.

#### Peptide Cleavage and Affinity Purification

Recombinant fusion proteins were purified using chitin affinity chromatography (New England Biolabs, Ipswich, MA, USA) by loading the clarified lysate of each recombinant fusion protein onto a column containing chitin resin. Following the manufacturer’s recommendations, the column was equilibrated with ice-cold column buffer. The clarified lysate was slowly dispensed onto the column at a flow rate of 0.1 mL/min to enhance efficient binding onto the chitin bed. The column was washed quickly with the column buffer at a higher flow rate to remove unbound proteins. The on-column cleavage of the recombinant fusion proteins was induced by incubating the resin with column buffer containing 50 mM DTT for 36 h at 4 °C. Cleavage buffer was removed, and the purified recombinant peptides were eluted with column buffer. The purified peptides were analyzed on a 4–20% tricine-SDS PAGE gel. After purification, DTT was removed from the buffer, and the protein was concentrated using a Millipore centricon tube. Peptide concentration was quantified using Bradford assay [63].

The confirmation of peptide cleavage and purification was performed by MALDI-TOF MS (Axima Confidence, Shimadzu, Japan) using 5 mg/mL α-Cyano-4-hydroxycinnamic acid (CHCA) as matrix. The samples were prepared by spotting 0.5 µL of the protein samples onto the MALDI metal plate, and immediately overlaying it with 0.5 µL of 5 mg/mL CHCA containing 1% (*v*/*v*) trifluoroacetic acid. The spots were left to dry and were placed in the instrument for analysis. The instrument parameter was set to MS mode; tuning was set to linear mode; laser power was set at 68; ion gate was set at 700 Da; pulsed extraction was set at 2330 bin.

### 4.6. Antimicrobial Activity of the Recombinant Peptides

#### 4.6.1. Minimum Inhibitory Concentrations of Recombinant Peptides

The broth microdilution method was employed to determine the minimum inhibitory concentrations (MICs) of the recombinant peptides against methicillin-resistant *S. aureus* (MRSA), *E. coli*, *K. pneumoniae*, *P. aeruginosa* and *C. albicans* [64,65]. The cell density was adjusted to 0.5 McFarland standard (10^6^ CFU/mL) in normal saline, and *C. albicans* were further diluted to 10^3^ CFU/mL in Mueller Hinton broth (Oxoid, Basingstoke, UK). Then, 10 μL of the inoculum suspension was added to 100 μL concentrations of recombinant defensin (3.75–985 µg/mL) or recombinant actifensin (11.5–2895 µg/mL), diluted in Mueller Hinton broth. Ampicillin was used as positive control for *E. coli*, *K. pneumoniae*, *P. aeruginosa*; vancomycin for MRSA; nystatin for *C. albicans.* The plates were incubated at 37 °C for 24 h for bacteria, or 48 h for *C. albicans*, then 30 μL of resazurin (0.015% *w*/*v*) was added to all wells and further incubated for 2–4 h to detect microbial activity by color change from blue to pink. The experiments were performed in triplicates, and the minimum inhibitory concentration was determined as the smallest concentration with no color change for each organism.

To determine the minimum bactericidal/fungicidal concentration (MBC/MFC), a loopful of inoculum from the wells without color change were plated on Mueller Hinton agar. The plates were then incubated at 37 °C for 24 h. The lowest concentration that showed no colonies was taken as the MBC/MFC [64,65].

#### 4.6.2. Synergistic Activity of Recombinant Peptides

The antimicrobial synergy of the recombinant peptides against the test organisms was assayed using the checkerboard method [66]. The fractional inhibition concentrations of recombinant defensin and recombinant actifensin against *P. aeruginosa* and *C. albicans* were determined. Recombinant defensin was diluted in a double fold horizontally, while recombinant actifensin was diluted vertically, such that all possible combinations of the respective concentrations were obtained. Then, 10 μL of the inoculum suspension was added into the respective wells. The plates were incubated at 37 °C for 24 h, or for 48 h for *C. albicans*, then 30 μL of resazurin (0.015%) was added to all wells and further incubated for 2–4 h to detect microbial activity by color change from blue to pink. The fractional inhibitory concentration index (FICI) was calculated as:

FICI = ΣFIC = ((MIC of defensin-d2 in combination/MIC of defensin-d2 alone) + (MIC of actifensin in combination/MIC of actifensin alone)).

The interactions were classified as being synergistic for ΣFIC values of ≤ 0.5, additive (≥ 0.5–1.0), indifferent (≥ 1.0–≤ 4.0) or antagonistic (ΣFIC > 4.0).

#### 4.6.3. Inhibitory Kinetics Analysis

The determination of the in vitro killing kinetics of the recombinant peptides against the test organisms was carried out [67], with modifications. A standardized inoculum of 1 × 10^6^ CFU/mL was used in the time-kill analysis through trypan-blue-based viable-colony counting using a Countess II counter (Invitrogen, Singapore). A control (untreated) sample (inoculum in MHB); samples treated with MIC, MIC ×2 and MIC ×4 of recombinant peptides; and samples treated with MIC, MIC ×2 and MIC ×4 of ampicillin, vancomycin and nystatin, respectively, were prepared. Samples were incubated at 37 °C for 24 h, or 48 h for *C. albicans*, and aliquots for viable counting were taken at 0.5, 1, 2, 4, 6, 12 and 24 h. A volume of 10 μL was taken from each sample, mixed with 10 μL of 0.4% trypan blue, and pipetted into a Countess chamber slide. The slide was inserted into the counter, and the number of viable cells was captured. Readings were performed in triplicates.

#### 4.6.4. Resistance Potential of Test Organisms to Recombinant Peptides

The investigation for the possible development of resistance in the sensitive strains was conducted by serial passaging, as described by Yang et al. [13], with modifications, for up to 14 days. The mid-log phase of the sensitive strains at 0.5 McFarland (10 μL per well) were inoculated into 100 μL MHB containing 0.5× the MIC of the recombinant peptides. After 16–24 h incubation at 37 °C, cells were reinoculated into 0.5× the MIC of the recombinant peptides, and this process was repeated four more times. After 6 passages of the cells in 0.5× the MIC, the cells were inoculated into the MIC of the recombinant peptides and incubated for 24–72 h. Where growth was observed, the cells were further inoculated into 2× the MIC of the recombinant peptides and incubated. Subsequent passaging into 4× the MIC and then 8× the MIC of the recombinant peptides was performed when growth was observed. The serial passaging was terminated when no visible growth was observed.

### 4.7. Hemolytic Activity of the Recombinant Peptides

Hemolytic activity of recombinant actifensin and defensin-d2 was assayed using fresh mouse erythrocytes [68]. The use of mouse blood in this study was approved by the Animal Care and Use Committee of Kenyatta University, Kenya (approval code: PKUA/003/E003). Mouse blood was aseptically withdrawn and centrifuged at 5000 rpm for 15 min to separate out the erythrocytes. The supernatant was removed, and 0.8% (*v*/*v*) of erythrocytes was prepared in sterile normal saline. Then, 100 μL of the erythrocytes was mixed with 100 μL concentrations of recombinant defensin (7.5–985 µg/mL) or recombinant actifensin (23–2895 µg/mL), and was incubated at 37 °C for 1 h. Erythrocytes treated with 1× PBS (pH) and 0.1% (*v*/*v*) Triton X-100 were used as negative and positive controls, respectively. After incubation, the samples were centrifuged at 5000 rpm for 10 min. The absorbance of the supernatant was measured at 540 nm. Percentage hemolysis was calculated as:

Hemolysis (%) = [(A_recombinant peptides_ − A_PBS_)/(A_Triton X-100_ − A_PBS_)] × 100

where A = absorbance.

### 4.8. Biofilm-Formation Inhibition by the Recombinant Peptides

To study the effect of recombinant actifensin and defensin-d2 on biofilm formation in *C. albicans* and *P. aeruginosa*, the test organisms (1 × 10^8^ CFU/mL) were grown in Saboraud’s Dextrose Agar or TSB medium in 96-well plates at 37 °C for 24 h, or 48 h for *C. albicans*, in the presence of 0.5–4 × the MICs of the peptides [13,69]. Ampicillin- and nystatin-treated cells were set as positive controls, while untreated cells were set as negative control. The plates were washed gently to remove the planktonic bacteria or yeast cells. Biofilms were stained for 30 min with 0.1% crystal violet, and excess stain was rinsed off with 1 × PBS. The dye bound to the adherent cells were resolubilized in 95% ethanol (200 μL/well), and absorbances were measured at 570 nm with a microplate reader (Synergy HTX reader, Agilent, Santa Clara, CA, USA). The percentage of biofilm inhibition was calculated as:

Biofilm inhibition (%) = [(A_treated_ – A_untreated_)/(A_untreated_)] × 100

where A = absorbance.

### 4.9. Peptide–Ligand Interaction of the Recombinant Peptides

A homology search using the peptide sequences was performed on the Protein Data Bank [70] (https://www.rcsb.org/search/advanced/sequence, accessed on 6 June 2022). The ‘basic search’ tool was used; then, a ‘search by sequences’ was performed using the peptide sequences as the query. The homologs generated as output were screened for characterized ligands. The ligand list was compiled, and their binding affinities assessed with the amino acid residues of both peptides using the Ligand Protein Interaction Comparison and Analysis online server [71] (https://webs.iiitd.edu.in/raghava/lpicom/index.php, accessed on 6 June 2022). The tool provided ligand-interacting residues as propensity scores of 0–9, with 9 being the maximum affinity score of a ligand to the interacting residue. The same set of ligands was used to screen both peptides to deduce similarities in ligand interactions between them. Prankweb [72,73] (https://prankweb.cz/, accessed on 6 June 2022) was used for the prediction of ligand-binding sites on the peptide 3D models.

### 4.10. Cell Permeability by the Recombinant Peptides

#### 4.10.1. Outer-Membrane-Permeabilization Assay

The outer-membrane permeability of the peptides was determined by using the N-phenyl-1-napthylamine (NPN) uptake assay [51]. *P. aeruginosa* and *C. albicans* were grown in MHB at 37 °C until OD_600_ reached 0.5. The cells were harvested by centrifugation at 11,000 rpm for 15 min, then washed in buffer (5 mM HEPES, 5 mM glucose, pH 7.4) at 650 rpm for 5 min. The cells were resuspended in 5 mM HEPES buffer to 0.2 at OD_600_. NPN (0.5 mM) was added to the cell suspension to a final concentration of 10 µM, and the background fluorescence was recorded (excitation λ = 350 nm, emission λ = 420 nm). Changes in fluorescence were recorded using a microplate fluorescence spectrophotometer (Synergy HTX reader, Agilent, Santa Clara, CA, USA). Peptide samples at 0.5 × MIC, 1 × MIC, 2 ×MIC and 4 × MIC were added to the wells, and fluorescence was recorded for 30 min at 5 min intervals, and at 60 min. Polymyxin B was used as a positive control because of its strong outer-membrane-permeabilizing properties [51]. Relative fluorescence was calculated by normalizing the fluorescence-intensity values to the untreated samples using the equation:

Relative fluorescence (RF) = (F_treated_)/(F_0_),

where F_treated_ is the fluorescence at a given peptide concentration, or of 10 µg/mL Polymyxin B; F_0_ is the fluorescence of NPN with the cells in the absence of peptide; normalized value = 1.

#### 4.10.2. Plasma-Membrane-Permeability Assay

To assess the plasma-membrane permeability of the peptides, a propidium iodide (PI) uptake assay was performed [74]. *P. aeruginosa* and *C. albicans* were cultured to mid-log phase in MHB. The cells were harvested by centrifugation at 11,000 rpm for 15 min, then washed in 1 × PBS (pH 7.2) at 650 rpm for 5 min. The cells were resuspended in the same buffer to an OD_600_ of 0.27. PI was added to 100 µL of the cell suspensions to a final concentration of 10 µg/mL, followed by an addition of peptide concentrations of 0.5 × MIC, 1 × MIC, 2 × MIC and 4 × MIC, accordingly. The suspension was incubated in the dark for 5 min. PI fluorescence was measured at excitation, and there were emission wavelengths of 580 and 620 nm, respectively, for 30 min at 5 min intervals, and at 60 min. Relative fluorescence of PI uptake was calculated using the equation:

Relative fluorescence (RF) = (F_treated_)/(F_0_),

where F_treated_ is the fluorescence at a given peptide concentration; F_0_ is the fluorescence of PI with the cells in the absence of peptide; normalized value = 1.

#### 4.10.3. Inner-Membrane-Depolarization Assay

The cytoplasmic-membrane-depolarization activity of the peptides was measured by using membrane-potential-sensitive fluorescent dye DiSC_3_(5) (3,3′-Dipropylthiadicarbocyanine Iodide), as described previously [51,52]. *P. aeruginosa* and *C. albicans* were grown in MHB at 37 °C until OD_600_ reached 0.5. The cells were harvested by centrifugation at 11,000 rpm for 15 min, then washed in buffer (5 mM sodium HEPES, 20 mM glucose, pH 7.4) at 650 rpm for 5 min. The cells were resuspended in the same buffer to an OD_600_ of 0.05. The cell suspensions were incubated in the dark for 30 min., with a final concentration of 0.4 µM DiSC_3_(5). Then, KCl was added to a final concentration of 0.1 M to equilibrate the cytoplasmic and external K+. The cell suspensions (200 µL) were dispensed into a 96-well plate, and the peptides were added to achieve 0.5 × MIC, 1 × MIC, 2 × MIC and 4 × MIC. Changes in fluorescence were recorded for 30 min at 5 min intervals, and at 60 min, using a fluorescence spectrophotometer (excitation λ = 622 nm; emission λ = 670 nm). Triton X-100 (0.1% *v*/*v*) was used as a positive control because of its strong depolarization activity. The relative fluorescence (RF) of the treated samples over the time series was calculated by normalization to the positive control using the equation:

Relative fluorescence (RF) = (F_treated_)/(F_0_),

where F_treated_ is the fluorescence at a given peptide concentration; F_0_ is the fluorescence of cells treated with Triton X-100 (0.1% *v*/*v*); normalized value = 1.

#### 4.10.4. Determination of Reactive-Oxygen-Species (ROS) Production

To measure differences in the ROS production between treated and untreated cells, *P. aeruginosa* and *C. albicans* were grown in MHB at 37 °C until OD_600_ reached 0.5 [75]. The cells were incubated with 10 μM of 2′,7′-dichlorodihydrofluorescein diacetate (H2DCFDA) for 1 h in the dark. After incubation, the cells were harvested by centrifugation at 11,000 rpm for 15 min, then washed twice with 1 × PBS (pH 7.2) at 650 rpm for 5 min. After, the cells were resuspended in the buffer to OD_600_ of 0.5, and 100 µL of the resultant cell suspension was dispensed into wells of 96-well plates, then treated with peptide concentrations of 0.5 × MIC, 1 × MIC, 2 × MIC and 4 × MIC, or 2 mM H_2_O_2_ as positive control, due to its strong oxidizing properties. Fluorescence measurements (excitation λ = 485 nm; emission λ = 535 nm) were taken for 30 min at 5 min intervals, and at 60 min. The untreated samples were used as negative controls. The relative fluorescence of the treated samples over the time series were calculated by normalization to the positive control (2 mM H_2_O_2_) using the equation:

Relative fluorescence (RF) = (F_treated_)/(F_0_),

where F_treated_ is the fluorescence at a given peptide concentration; F_0_ is the fluorescence of cells treated with 2 mM H_2_O_2_; normalized value = 1.

## 5. Conclusions

We report the recombinant production of the potent broad-spectrum antimicrobial peptides, spinach defensin and actifensin, from *Actinomyces ruminicola* in an *E. coli* system. The recombinant peptides, recovered in high yields and purities, exhibited remarkable antifungal and antibacterial activity against MDR *C. albicans* and *P. aeruginosa*, with no resistance potential, and they showed low toxicity. We also conclude that the mechanisms of action of these peptides against MDR *C. albicans* and *P. aeruginosa* is through membrane permeability and oxidative stress, which could further affect essential metabolic pathways, thereby resulting in cell death.

## Figures and Tables

**Table 1:** In silico characterization of the peptides.

Source	Peptide	Amino Acid Sequence	Length	MW * (Da)	pI *	Net Charge	GRAVY	II *	Signal Peptide	AI *
*Spinacia oleracea*	Defensin-d2	GIFSSRKCKTPSKTFKGICTRDSNCDTSCRYEGYPAGDCKGIRRRCMCSKPC	52 aa	5809.73	9.3	+7.6	−0.810	55.68 (unstable)	+	24.42
*Actinomyces ruminicola*	Actifensin	GFGCNLITSNPYQCSNHCKSVGYRGGYCKLRTVCTCY	37 aa	4097.70	8.89	+3.8	−0.243	8.22 (stable)	+	47.30

* MW—molecular weight; pI—isoelectric point; II—instability index (values above 40 are considered unstable); AI—aliphatic index.

**Table 2:** Minimum inhibitory/bactericidal/fungicidal concentrations of recombinant actifensin and defensin-d2.

Test Organism	MIC (µg/mL)					MBC/MFC (µg/mL)	
	Actifensin	Defensin-d2	Ampicillin	Nystatin	Vancomycin	Actifensin	Defensin-d2
MRSA	23	-	NA	NA	4	-	ND
*E. coli*	-	30	5000	NA	NA	ND	246
*P. aeruginosa*	1448	7.5	10,000	NA	NA	1448	123
*K. pneumoniae*	-	30	5000	NA	NA	ND	-
*C. albicans*	23	7.5	NA	1290	NA	724	63

NA—antibiotics not applicable to organism; ND—not determined; - indicates no MIC or MBC. Values presented are means of triplicate data, and standard deviation is 0.00.

**Table 3:** Synergistic activity of recombinant peptides.

Test Organism	Actifensin Alone (µg/mL)	Actifensin Combination (µg/mL)	Defensin-d2 Alone (µg/mL)	Defensin-d2 Combination (µg/mL)	FIC Index Value	Remark
*P. aeruginosa*	1448	1448	7.5	63	>4	Antagonistic
*C. albicans*	23	181	7.5	63	>4	Antagonistic

FIC—fractional inhibitory concentration. Values presented are means of triplicate data, and standard deviation is 0.00.

**Table 4:** Nucleotide sequences of primers used in this study.

Tag	Sequences 5′–3′	Target	Amplicon Size (bp)
Primers for colony PCR			
ptxb1_F	AACTGCCAGGAATTGGGGAT	pTXB1	730
ptxb1_R	GCTTCCAAGAAACGCACCAG		
Dfn_F	AGTGTAAGACTCCTTCTAAGACTTT	defensin	102
Dfn_R	CCTTACAATCACCAGCAGGGT		
Afn_F	CTTCGGCTGCAACCTCATCA	actifensin	103
Afn_R	GGTGCAGACCGTCCGAAG		
Primers for cloning			
Def_F	AAGAAGGAGATATACGGTATTTTTTCTTCTAGAAAGTGTAAGACTCCTTCTAAGAC	defensin	184
Def_R	TCTCCCGTGATGCAGACAAGGCTTAGAACACATACATCTTCTAATACC		
Acti_F	AAGAAGGAGATATACGGCTTCGGCTGCAACCT	actifensin	141
Acti_R	ATCTCCCGTGATGCAGTAGCAGGTGCAGACCGT		
Vector_F	TGCATCACGGGAGATGCACT	pTXB1	6654
Vector_R	GTATATCTCCTTCTTAAAGTTAAACAAAATTATTTCTAGAGGGGA		

## Data Availability

Not applicable.

## Supplementary Materials

The following supporting information can be downloaded at: https://www.mdpi.com/article/10.3390/molecules27144325/s1, Figure S1: Gel electrophoresis of colony PCR amplification of gene and plasmid fragments; Figure S2: Alignment of actifensin ORF (A) and defensin-d2 ORF (B) with respective sequences of recombinant pTXB1 after plasmid sequencing; Table S1: Peptide-ligand affinity scores of defensin-d2 and actifensin.

## Sample Availability

Samples of the peptides are available from the authors on reasonable request.
