# Supplementary material for: Membrane Permeabilization and Antimicrobial Activity of Recombinant Defensin-d2 and Actifensin against Multidrug-Resistant Pseudomonas aeruginosa and Candida albicans
Source: Molecules. 2022 Jul 6;27(14):4325. doi: 10.3390/molecules27144325 (PMC9317813; doi:10.3390/molecules27144325)
Supplement: Supplementary file 1 [file molecules-27-04325-s001.zip › molecules-1781231-supplementary.pdf]

Supplementary data

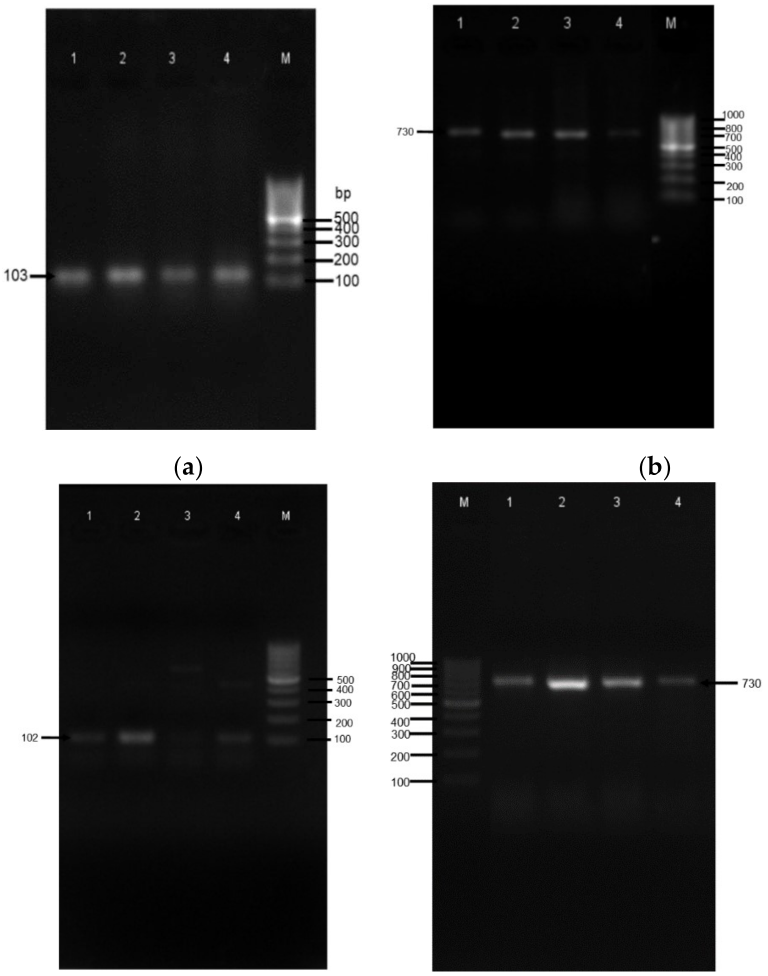

(**a**) (**b**)

(**c**) (**b**)(**d**)

Figure S1. Gel electrophoresis of colony PCR amplification of gene and plasmid fragments.

(**a**) colonies positive for actifensin gene, L1 – 4, M-100 bp ladder; (**b**) actifensin-positive clones showing the presence of pTXB1, L1 – 4, M-100 bp ladder; (**c**) colonies positive for defensin-d2 gene, L1 – 4, M-100 bp ladder; (**d**) actifensin-positive clones showing the presence of pTXB1, L1 – 4, M-100 bp ladder.

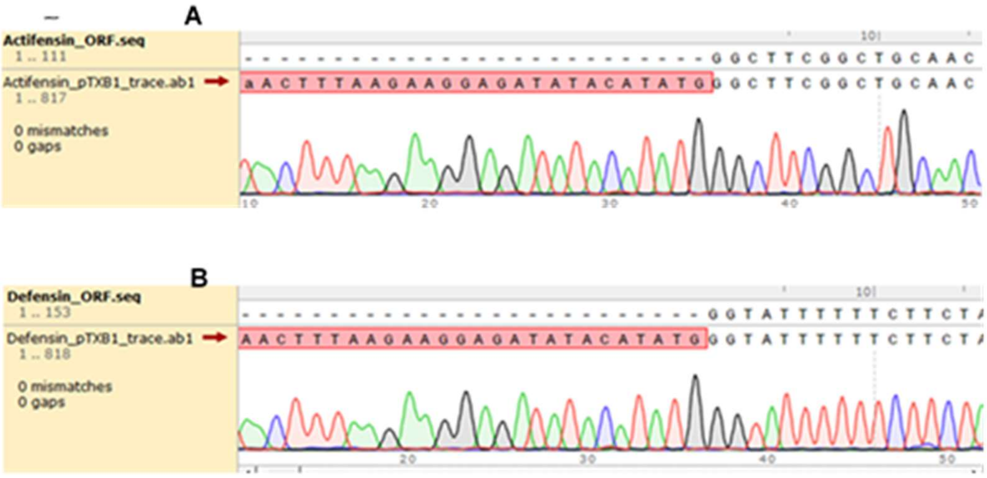

Figure S2. Alignment of actifensin ORF (A) and defensin-d2 ORF (B) with respective sequences of recombinant pTXB1 after plasmid sequencing. The alignment shows an in-frame insertion of both target genes at the start codon (ATG)

Table S1. Peptide-ligand affinity scores of defensin-d2 and actifensin

	**Defensin-d2**			**Actifensin**	
Ligand symbol	Residues	Propensity score	Ligand symbol	Residues	Propensity score
ACT	F<br>R<br>Y	5<br>8<br>6	ACT	F<br>R<br>Y<br>H	5<br>8<br>6<br>9
PO_4	R	8	PO_4	R<br>H	8<br>9
EDO	F<br>R<br>K<br>N<br>Y	6<br>7<br>4<br>5<br>9	EDO	F<br>R<br>K<br>N<br>Y<br>H	6<br>7<br>4<br>5<br>9<br>5
PA8	-	-	PA8	-	-
PGE	R<br>Y	5<br>6	PGE	R<br>Y	5<br>6
MRD	F<br>R<br>Y<br>M	6<br>5<br>8<br>5	MRD	F<br>R<br>Y	6<br>5<br>8
P10	R<br>I<br>K	5<br>4<br>9	P10	R<br>I<br>K<br>H	5<br>4<br>9<br>6
44E	-	-	44E	-	-
SO_4	R	9	SO_4	R	9

	K	4		K<br>H	4<br>6
GOL	F<br>R<br>N<br>D	4<br>6<br>4<br>4	GOL	F<br>R<br>N<br>Y<br>Q<br>H	4<br>6<br>4<br>6<br>4<br>6
CL	R<br>K<br>N	9<br>5<br>5	CL	R<br>K<br>N<br>H	9<br>5<br>5<br>9
FLC	R<br>K	8<br>4	FLC	R<br>K<br>H	8<br>4<br>9
PEG	R<br>Y	5<br>6	PEG	R<br>Y<br>H	5<br>6<br>5
13C	R<br>Y	6<br>5	13C	R<br>Y<br>N<br>H	6<br>5<br>4<br>9
